# Diversity and taxonomic differences in the oral microbiota of stroke patients: a systematic review and meta-analysis

**DOI:** 10.3389/fmicb.2026.1874193

**Published:** 2026-07-15

**Authors:** Yinlian Chen, Yunxue Tian, Yingju Jin, Xueqin Wu, Xiaomei Li, Wei Du, Wuanqin Li, Juan Li

**Affiliations:** 1 School of Nursing, Zunyi Medical University, Zunyi, China; 2 School of Nursing, Guizhou Medical University, Guiyang, China; 3 School of Nursing, Guizhou University of Traditional Chinese Medicine, Guiyang, China; 4 Department of Nursing, Guizhou Provincial People’s Hospital, Guiyang, China

**Keywords:** stroke, oral microbiota, dysbiosis, systematic review, meta-analysis

## Abstract

**Background:**

In recent years, a growing body of evidence suggests that stroke may be associated with an imbalance in the oral microbiome. To further elucidate this potential link, this study aims to systematically compare differences in the oral microbiome between stroke patients and healthy individuals.

**Objective:**

To systematically evaluate the differences in oral microbiota diversity and taxonomic composition between stroke patients and healthy controls.

**Method:**

From the date each database was established up to 20 February 2026, we conducted searches in CNKI, Wanfang, PubMed, Embase, SinoMed, Web of Science, the Cochrane Library and grey literature databases, with the aim of identifying studies reporting on the oral microbiota of stroke patients and healthy individuals. The Newcastle-Ottawa Scale (NOS) was used to assess the risk of bias in the included studies, and meta-analysis was performed using RevMan 5.4 software. The pooled effect size was calculated as the standardized mean difference (SMD) and a parallel Z-test was conducted; heterogeneity was assessed using Cochran’s Q test and the I^2^ statistic.

**Result:**

This meta-analysis of 11 studies (746 stroke patients, 552 controls) showed upward trends in Observed species (SMD = 0.39, 95% CI: 0.05–0.72, I^2^ = 84%) and Shannon (SMD = 0.31, 95% CI: 0.02–0.61, I^2^ = 82%) indices, though these findings were not robust in sensitivity analyses. Chao1 and Simpson showed no significant differences. Eight of nine *β*-diversity studies reported significant differences between groups. Meta-analysis showed higher Bacteroidota (SMD = 0.36, 95% CI: 0.18–0.54, 3 studies; I^2^ = 44%). Higher abundances of *Firmicutes, Spirochaetes*, and several genera were suggested by descriptive synthesis but should be considered exploratory.

**Conclusion:**

The oral microbiota of stroke patients exhibits characteristic changes, which may provide preliminary clues for understanding post-stroke oral microbial alterations and offer a theoretical basis for oral care, but require validation in prospective studies.

**Systematic review registration:**

Unique Identifier: CRD420251235256, https://www.crd.york.ac.uk/PROSPERO/view/CRD420251235256.

## 1 Introduction

Stroke is a condition affecting the central nervous system of the brain that is highly widespread, causes substantial impairment, and has high mortality rates (GBD 2019 Stroke Collaborators, 2021). Consequently, it poses a major public health challenge, severely endangering human health and placing heavy burdens on individuals, families, and society at large (Zhao et al., 2023). The pathogenesis of stroke and its related risk factors are highly intricate (Ren et al., 2020; Kuriakose and Xiao, 2020). Even though conventional risk factors like diabetes, dyslipidemia, and hypertension are well-known and frequently managed (Boehme et al., 2017), they do not fully explain all causes of stroke, suggesting that other factors may also be involved.

Recently, a link between the oral microbiome and stroke has gotten more attention. The oral cavity is the second largest microbial reservoir in the human body after the gut (Wade, 2013). Dysbiosis of oral microbiota can lead to chronic infectious illnesses, like tooth decay and gum disease (Lamont et al., 2018; Rajasekaran et al., 2024; Xu et al., 2023), and accumulating evidence indicates that periodontal disease is a significant risk factor for stroke (Sen et al., 2017). A study conducted in stroke patients has demonstrated that certain species within the *Streptococcus* genus—particularly *Streptococcus salivarius*—are the most prevalent type of bacteria found in the mouth in the initial stages following a stroke (Boaden et al., 2017). Another case–control study also showed that *Streptococcus mutans* bacteria were more frequently detected in stroke patients’ saliva samples compared with healthy controls (Inenaga et al., 2018). Furthermore, stroke-related functional impairments (such as difficulty brushing teeth, chewing, and swallowing) may further compromise oral hygiene and contribute to an imbalance in the oral microbiome (Lam et al., 2013; Dai et al., 2017). Overall, available evidence indicates that individuals with stroke exhibit changes in their oral microbial composition.

16S rRNA sequencing and high-flux gene tools are widely used in this field. But the results from current studies are not the same. For germ diversity, some studies found that stroke patients have a big increase in mouth germ *α*-diversity (like Shannon and Chao1 numbers) compared to healthy people (Yao et al., 2025; Sun et al., 2023). In contrast, other studies failed to detect a statistically significant change in *α*-diversity between the two groups (Zhao et al., 2025; Zheng et al., 2026). With regard to taxonomic composition, the relative abundances of the *Firmicutes* and *Bacteroidetes* phyla have increased in some studies (Sun et al., 2023; He et al., 2024), while they have decreased in others (Wang et al., 2022; Chang, 2024). Consequently, there is currently a lack of quantitative synthesis regarding differences in the oral microbiome between stroke patients and healthy controls; existing studies remain inconsistent regarding the direction and extent of these differences, and there is an urgent need for a quantitative assessment through a meta-analysis.

Elucidating the diversity and taxonomic characteristics of the oral microbiome in stroke patients may provide insights into post-stroke oral microbial alterations and offer a theoretical basis for developing targeted oral care strategies for this population. Consequently, this study aims to quantitatively compare differences in oral microbiome diversity (*α*-diversity and *β*-diversity) and taxonomic composition (at the phylum and genus levels) between stroke patients and healthy controls through a systematic review and meta-analysis.

## 2 Methods

### 2.1 Search for literature

This systematic review and meta-analysis were registered in PROSPERO (CRD420251235256) and conducted in accordance with the PRISMA guidelines. We searched the following electronic databases from inception to February 20, 2026: Wanfang, CNKI, PubMed, EMBASE, SinoMed, Web of Science, and the Cochrane Library. The search terms were primarily based on MeSH subject headings, supplemented by some free-text terms; search queries were constructed using the Boolean operators ‘OR’ and ‘AND’, and adjusted according to the characteristics of each database. The search strategy for English databases is exemplified by PubMed, and the search strategy for Chinese databases is exemplified by Wanfang. The detailed search terms and expressions are provided in the Supplementary Tables 1, 2. The search was restricted to Chinese and English articles, and only studies with available full text were included. To identify grey literature, we searched the OpenGrey database and Google Scholar; after screening, no eligible studies were identified from these sources. In addition, we manually checked the reference lists of all eligible studies to maximize the comprehensiveness of the literature retrieval. This review included original research papers that compared oral microbiota composition between stroke patients and healthy controls. Two review authors independently screened the titles, abstracts, and full texts. Any disagreements were resolved by discussion or by consulting a third reviewer.

### 2.2 Eligibility criteria

Two authors (CYL and TYX) independently conducted the literature screening and full-text review, and included eligible studies based on predefined criteria. The specific inclusion criteria are as follows: (1) Study population: Studies must include both a stroke group and a healthy control group. The stroke group comprised patients with clinically or radiologically confirmed stroke; no restrictions were placed on stroke subtypes, including all types such as ischemic stroke and hemorrhagic stroke. The healthy control group consisted of healthy adults with no history of stroke, and was comparable to the stroke group in terms of baseline characteristics such as age and gender; (2) Sample type: Oral microbiome data must be derived from oral samples, including saliva, gingival crevicular fluid, subgingival plaque or oropharyngeal swabs; (3) Age: Study participants must be adults (aged ≥18 years); studies involving children are excluded, as their microbial composition is unstable during development and cannot be compared with that of adults; (4) Outcome measures: Studies must report oral microbiome diversity (*α*-diversity or *β*-diversity) or relative abundance (at the phylum or genus level), and provide sufficient statistical data (e.g., mean, standard deviation, interquartile range, *p*-value, minimum, maximum, etc.) to calculate effect sizes; (5) Exclusion criteria: Case reports, systematic reviews and animal studies are excluded.

### 2.3 Measures of results

Alpha diversity, beta diversity, and proportional abundance were used to compare the oral microbiota of stroke patients and healthy controls.

### 2.4 Extraction of data and evaluation of quality

Two authors (CYL and TYX) independently extracted and assessed the data; any discrepancies were resolved through discussion between the two authors or by consulting a third researcher. From the remaining studies, the following information was extracted: first author, title of the article, year of publication, country of origin, participant age range, sample size, type of oral specimen, microbial assessment method, targeted 16S rRNA gene region for sequencing, and reported stroke-related oral microbiota alterations. To assess changes in the relative abundances of microbial communities at the phylum and genus levels, trends in the increase or decrease in the relative abundances of seven and six major microbial taxonomic units, respectively, were identified. Furthermore, at the phylum level, standard deviations (SD) were reported for only four bacterial taxonomic units across two or more studies. For data presented in graphical form (such as box plots or bar charts) without specific numerical values being reported directly, we used the GetData Graph Digitizer software (version 2.26) to extract the interquartile range, maximum and minimum values from the graphs, and calculated the mean and standard deviation based on these values.

Using the Newcastle-Ottawa Scale (NOS), the included observational studies’ risk of bias was assessed. Two independent researchers assessed each study for potential biases arising from study design, implementation, and result analysis. The assessment considered three domains: (1) Check how people were chosen. This means to see if the way to find cases, if the cases stand for the whole group, how the controls were picked, and how the controls were confirmed are all right. (2) How well the cases and controls can be compared. This is judged by the study design and the way of analyzing numbers. (3) How the exposure was measured. This includes whether the way to measure exposure is true and steady, if the same way was used for cases and controls, and the rate of people who did not answer. The NOS scoring criteria are as follows: 0–3 points: high risk of bias, 4–6 points: moderate risk of bias, 7–9 points: low risk of bias.

### 2.5 Meta-analysis

The pooled effect size was calculated using the random-effects inverse-variance-weighted model in RevMan 5.4 software. For data deviating from a normal distribution, the mean and standard deviation were estimated from the median, maximum, and minimum values using a transformation formula reported in the literature (Hozo et al., 2005). The effect size for continuous variables was expressed as Hedges’ g, calculated as the difference between the means of the stroke group and the healthy control group divided by the pooled standard deviation of the two groups. Heterogeneity among studies was assessed using the Q statistic and the I^2^ statistic. Publication bias was evaluated qualitatively using funnel plots. The significance level for all statistical tests was set at *p* < 0.05. Regarding data presentation, the characteristics and quality assessment results of individual included studies were presented in tabular form; in the summary results, the pooled effect size and its 95% confidence interval were presented using a forest plot.

### 2.6 Availability of data

To obtain the necessary data, the lead author was contacted directly.

## 3 Results

### 3.1 Study inclusion and features

**Figure 1:**
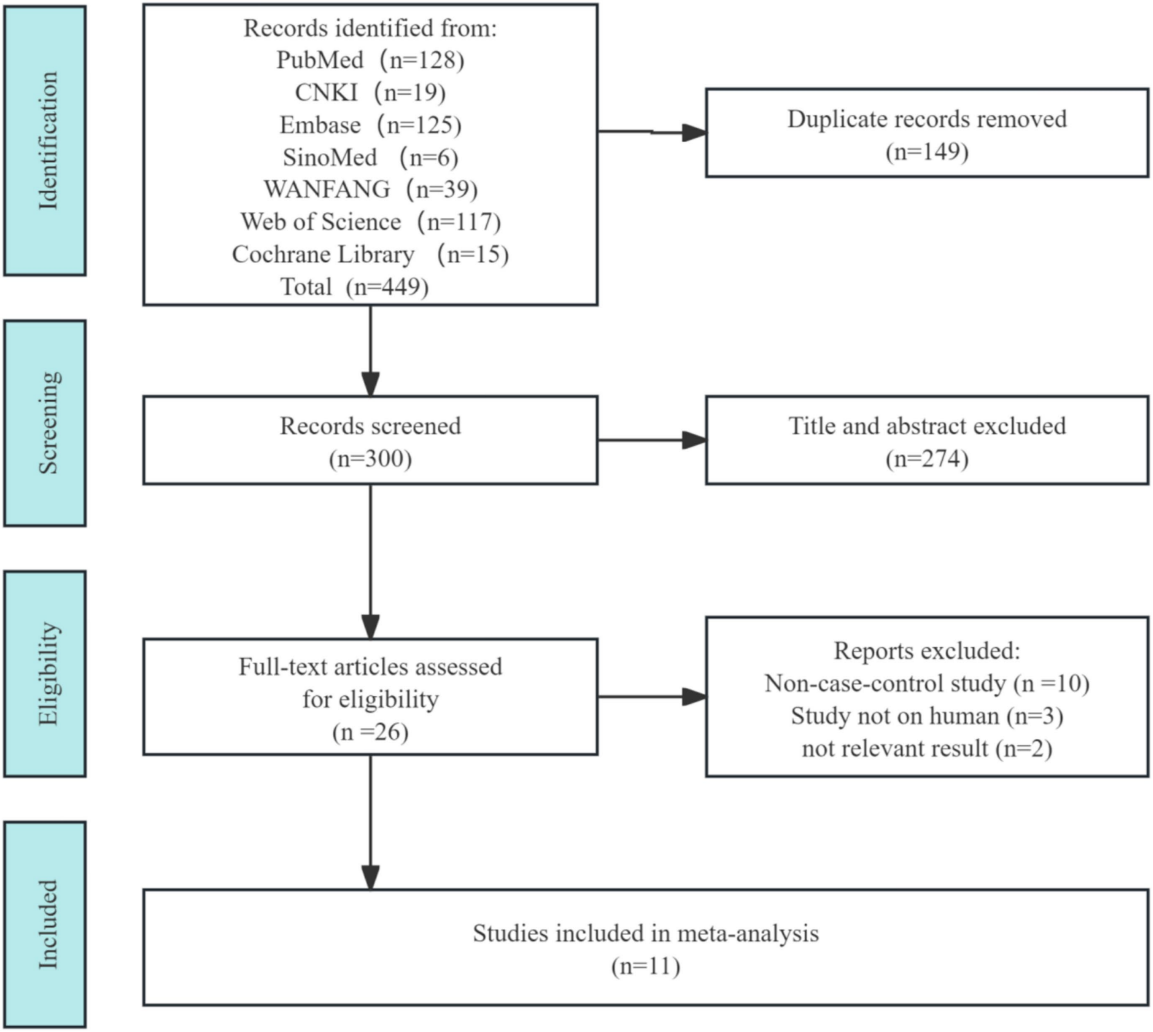
The study selection procedure is depicted in the PRISMA flow.

The flowchart illustrating the literature search and screening process is shown in Figure 1. From seven databases, 449 relevant articles were found, of which 149 were duplicates. Of the articles screened by title and abstract, 274 were excluded for not meeting the eligibility criteria; among the remaining, further exclusions included 10 studies without healthy controls, 3 involving non-human subjects, and 2 that failed to report the necessary outcome measures. Ultimately, include 11 article accords with a condition into the meta-analysis (Yao et al., 2025; Sun et al., 2023; Zhao et al., 2025; Zheng et al., 2026; He et al., 2024; Wang et al., 2022; Chang, 2024; Huang, 2024; Yang, 2022; Manzoor et al., 2025; Manzoor et al., 2024). A total of 1,298 participants were included (746 stroke patients and 552 healthy controls), comprising five Chinese-language studies and six English-language studies. Table 1 displays the specific features of the listed studies.

**Table 1:** Baseline features of selected studies.

First author	Year	Country	Age (case/control)	Sample size (case/control)	Oral microbiota assessment	Oral sample	Collection time
Tianyu Chang	2024	China	57.40 ± 7.10/56.00 ± 8.60	30/30	16S rRNA sequencing (V3-V4)	Saliva	3d
Zhiyan Huang	2024	China	65.00 ± 11.70/63.00 ± 13.00	100/31	16S rRNA sequencing (V1-V9)	Mucosal swab	24 h
Mengjia Yang	2022	China	54.20 ± 8.80/56.70 ± 3.20	52/26	16S rRNA sequencing (V4)	Gingival sulcus- swab sample	3mo.
Xiaohua Zhao	2025	China	63.36 ± 11.65/54.50 ± 7.62	14/8	16S rRNA sequencing (V3-V4)	Saliva	7d
Lihe Yao	2025	China	59.69 ± 11.53/63.08 ± 12.95	32/25	16S rRNA sequencing (V1-V9)	Subgingival plaque	48 h
Huidi Wang	2022	China	53.90 ± 8.80/57.00 ± 8.80	47/34	16S rRNA sequencing (V4)	Gingival sulcus- swab sample	3mo.
Wenbo Sun	2023	China	57.00 ± 7.90/57.00 ± 8.60	52/46	16S rRNA sequencing (V3-V4)	Saliva	24 h
Muhammed Manzoor	2025	Multicenter	39.40 ± 7.70/40.10 ± 7.60	134/138	16S rRNA sequencing (V1-V2)	Subgingival plaque sample	case:112d control:68d
Qiuxing He	2025	China	59.52 ± 5.02/58.71 ± 3.74	30/30	16S rRNA sequencing	Throat swab	24 h
Muhammed Manzoor	2024	Multicenter	41.98 ± 2.32/41.67 ± 2.15	155/153	Shotgun metagenomic sequencing	Saliva	NR
Xiaohong Zheng	2026	China	63.13 ± 8.29/62.06 ± 6.83	100/31	16S rRNA sequencing	Saliva	24 h

### 3.2 Quality assessment

The Newcastle-Ottawa Scale (NOS) was used to assess the quality of the 11 included case–control studies. According to the NOS scoring criteria (0–3 points indicating a high risk of bias, 4–6 points indicating a moderate risk of bias, and 7–9 points indicating a low risk of bias), the results showed that the total NOS scores for the 11 studies ranged from 7 to 8 points, with an average score of 7.6 points. Of these, 3 studies (27.3%) scored 7 points, and 8 studies (72.7%) scored 8 points. All 11 studies (100%) were assessed as having a low risk of bias. The specific scoring results are shown in Table 2.

**Table 2:** NOS-based quality appraisal of the included studies.

Study included	Study design	Newcastle-Ottawa Scale			Overall quality assessment
		Selection	Comparability	Outcome	
Chang (2024)	Case–control	4	1	3	8
Huang (2024)	Case–control	4	1	3	8
Yang (2022)	Case–control	3	1	3	7
Zhao et al. (2025)	Case–control	4	1	3	8
Yao et al. (2025)	Case–control	3	1	3	7
Wang et al. (2022)	Case–control	4	2	2	8
Sun et al. (2023)	Case–control	3	2	3	8
Manzoor et al. (2025)	Case–control	3	2	2	7
He et al. (2024)	Case–control	3	2	3	8
Manzoor et al. (2024)	Case–control	3	2	3	8
Zheng et al. (2026)	Case–control	4	1	3	8

### 3.3 Certainty of evidence assessment

The GRADE system was used to assess the certainty of evidence for the primary outcome measures. As all included studies were observational studies, the starting certainty level was low (⊕ ⊕ ○○). The evidence was assessed for downgrading based on five domains: risk of bias, inconsistency, indirectness, imprecision, and publication bias. The certainty of evidence was classified into four levels: high (⊕⊕⊕⊕), moderate (⊕⊕⊕○), low (⊕⊕○○), and very low (⊕○○○).

### 3.4 Diversity of *α* and *β*

**Figure 2:**
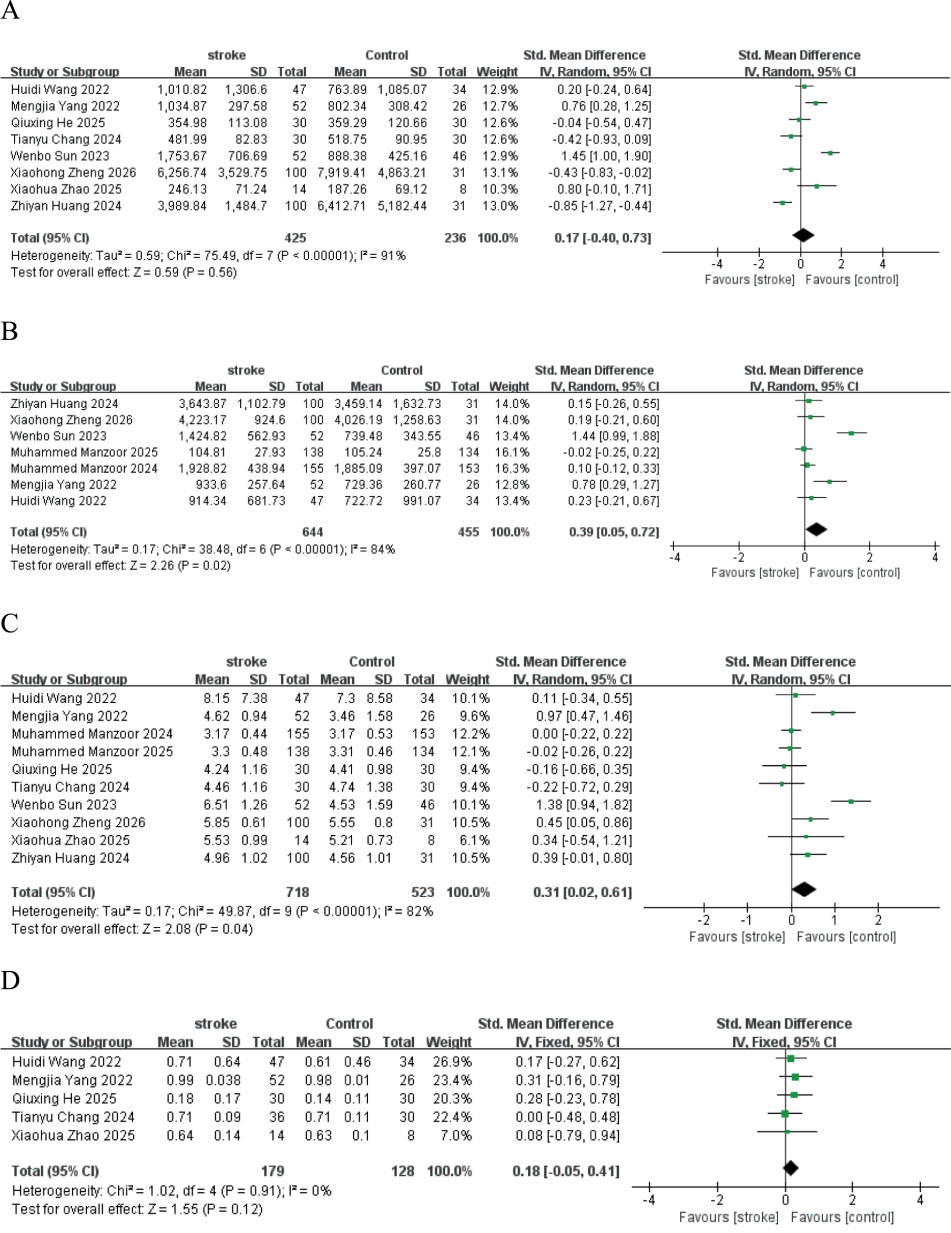
Alpha-diversity indices in stroke vs. control groups. Chao1 **(A)**, observed species **(B)**, Shannon index **(C)**, and Simpson index **(D)** forest plots that compare alpha-diversity indices between the stroke group and the control group.

Both species richness—the quantity of unique species found in a community—and species evenness—the degree to which individuals are dispersed equally among those species—are measured by the alpha diversity index. Alpha diversity is commonly quantified using the Chao1 index, Shannon index, Simpson index, and observed species index. The results demonstrated statistically significant increases in both the Observed Species index (*n* = 7; SMD = 0.39; 95% CI: 0.05 to 0.72; I^2^ = 84%) (Figure 2B) and the Shannon index (*n* = 10; SMD = 0.31; 95% CI: 0.02 to 0.61; I^2^ = 82%) (Figure 2C). The Chao1 index (*n* = 8; SMD = 0.17; 95% CI: −0.40 to 0.73; I^2^ = 91%) (Figure 2A) and the Simpson index (n = 5; SMD = 0.18; 95% CI: −0.05 to 0.41; I^2^ = 0%) (Figure 2D) revealed no statistically significant difference between the stroke group and the control group.

The *β*-diversity index quantifies species turnover or compositional dissimilarity among ecosystems, reflecting differences in taxonomic composition between ecological communities. Of the 11 included studies, nine evaluated β-diversity (Table 3), while the remaining two did not (Wang et al., 2022; Chang, 2024). Of the nine studies assessing β-diversity, eight (88.9%) reported statistically significant intergroup differences. Bray–Curtis dissimilarity-based principal coordinate analysis was the most used technique; Seven studies found significant variations in the microbial community structure between stroke patients and healthy control subjects (Huang, 2024; Yang, 2022; Zhao et al., 2025; Yao et al., 2025; Manzoor et al., 2025; He et al., 2024; Zheng et al., 2026), whereas one study found no statistically significant difference (Manzoor et al., 2024). Huang (2024) noticed that the results of principal coordinate analysis (PCoA) based on Bray–Curtis dissimilarity, PCoA using unweighted UniFrac distance, and PCoA using weighted UniFrac distance all revealed notable variations among stroke victims and healthy controls. Muhammed Manzoor (26) found that the results of principal coordinate analysis (PCoA) based on Bray–Curtis dissimilarity, PCoA using unweighted UniFrac distances, and PCoA using Jaccard distances all revealed that the stroke group and the healthy control group did not differ significantly.

**Table 3:** Summarizes the evaluations of beta diversity in the included research.

Study	β diversity	Findings	Statistic value
Huang (2024)	PCoA based on Bray–Curtis dissimilarity	A significant difference in oral microbial composition among S and H	*p* < 0.01
	PCoA based on the Unweighted UniFrac distances	A significant difference in oral microbial composition among S and H	*p* < 0.01
	PCoA based on the Weighted Unifrac distance	A significant difference in oral microbial composition among S and H	*p* < 0.01
Yang (2022)	PCoA based on Bray–Curtis dissimilarity	A significant difference in oral microbial composition among S and H	*p* = 0.005
Zhao et al. (2025)	PCoA based on Bray–Curtis dissimilarity	A significant difference in oral microbial composition among S and H	*p* = 0.002
Yao et al. (2025)	PCoA based on Bray–Curtis dissimilarity	A significant difference in oral microbial composition among S and H	*p* = 0.001
Sun et al. (2023)	PCoA based on the Unweighted UniFrac distances	A significant difference in oral microbial composition among S and H	*p* < 0.001
Manzoor et al. (2025)	PCoA based on Bray–Curtis dissimilarity	A significant difference in oral microbial composition among S and H	*p* = 0.003
	PCoA analysis based on Jaccard distance	A significant difference in oral microbial composition among S and H	*p* = 0.007
He et al. (2024)	PCoA based on Bray–Curtis dissimilarity	A significant difference in oral microbial composition among S and H	*p* = 0.003
Manzoor et al. (2024)	PCoA based on Bray–Curtis dissimilarity	No difference in oral microbial composition among S and H	*p* = 0.325
	PCoA based on Jaccard distance	No difference in oral microbial composition among S and H	*p* = 0.850
	PCoA based on the Weighted Unifrac distance	No difference in oral microbial composition among S and H	*p* = 0.403
Zheng et al. (2026)	PCoA based on Bray–Curtis dissimilarity	A significant difference in oral microbial composition among S and H	*p =* 0.001

### 3.5 Microbial taxa’s relative abundance

This review used both descriptive synthesis and meta-analysis to present microbial changes; conclusions are based primarily on meta-analysis results, with descriptive synthesis as supplementary.

**Figure 3:**
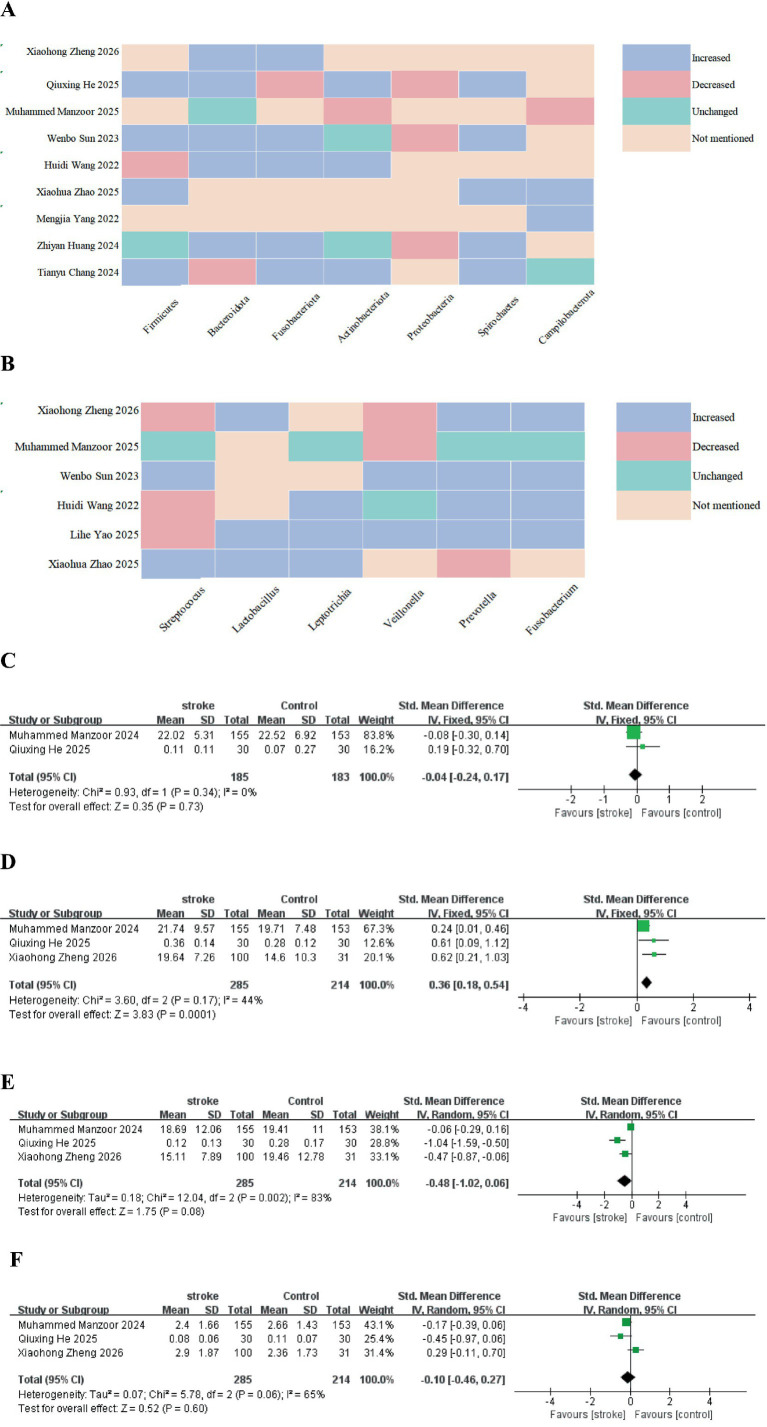
Oral microbiota abundance: stroke vs. healthy controls. **(A)** Phylum-level heatmap; **(B)** gender-stratified heatmap; **(C–F)** forest plots depicting differences in relative abundance of *Actinomycota* **(C)**, *Bacteroidota* **(D)**, *Proteobacteria* **(E)**, and *Fusobacteriota* **(F)** between the control group that was healthy and the stroke group.

To compare the relative abundances of representative bacterial phyla between stroke patients and healthy controls, nine studies were considered. Descriptive synthesis showed that, in the group of stroke, the relative abundance of *Firmicutes* (66.67%, 4/6), *Bacteroidota* (71.43%, 5/7), *Fusobacteriota* (83.33%, 5/6), and *Spirochaetes* (100%, 5/5) was higher. In contrast, *Proteobacteria* exhibited a consistent decrease across all three included studies (100%, 3/3 studies) (Figure 3A).

Meta-analysis, according to the results of the stroke group of *Bacteroidota,* significantly increased abundance than healthy controls (n = 3, SMD = 0.36, 95% CI: 0.18 to 0.54, I^2^ = 44%) (Figure 3D). No statistically significant differences were observed for the abundances of the other bacterial phyla included in the analysis. Specifically, *Actinomycetota* was assessed in two studies (n = 2; SMD = −0.04; 95% CI: −0.24 to 0.17; I^2^ = 0%) (Figure 3C); *Proteobacteria* was evaluated in three studies (n = 3; SMD = −0.48; 95% CI: −1.02 to 0.06; I^2^ = 83%) (Figure 3E); and *Fusobacteriota* was also reported in three studies (n = 3; SMD = −0.10; 95% CI: −0.46 to 0.27; I^2^ = 65%) (Figure 3F).

Examining how the stroke group and the healthy control group differed in representative microbial genera, based on six included studies. Descriptive synthesis showed that the relative abundances of *Lactobacillus* (100%, 3/3), *Leptotrichia*(75%, 3/4), *Prevotella* (66.67%, 4/6), and *Fusobacterium* (80%, 4/5) were higher in the stroke group (Figure 3B). These genus-level findings are based on descriptive synthesis and have not been quantitatively validated; they should be regarded as exploratory rather than definitive conclusions.

### 3.6 Sensitivity analysis

The one-by-one exclusion approach sensitivity analysis showed that eliminating (Sun et al., 2023), the heterogeneity of the Chao1 index (see Supplementary Figure 1A), observed species (see Supplementary Figure 1B) and Shannon index (see Supplementary Figure 1C) all decreased significantly (I^2^ fell from 91 to 82%, from 84 to 52% and from 82 to 62% respectively). The pooled SMD for Observed species decreased from 0.39 (95% CI: 0.05–0.72) to 0.13 (95% CI: −0.06 to 0.33), and for Shannon index from 0.31 (95% CI: 0.02–0.61) to 0.18 (95% CI: −0.04 to 0.40), with both losing statistical significance, suggesting that this study was the primary source of heterogeneity and that the significance of these findings was heavily dependent on this single study. Notably, this study enrolled only ischemic stroke patients with two subtypes: large-artery atherosclerosis (LAA) and small-artery occlusion (SAO). In addition, after excluding the study by Manzoor et al. (2024), heterogeneity was reduced for both *Bacteroidota* (Supplementary Figure 2A; I^2^ decreased from 44 to 0%) and *Pseudomonadota* (Supplementary Figure 2B; I^2^ decreased from 83 to 64%), and the direction of the pooled estimates remained unchanged and both remained statistically significant (Bacteroidota: SMD 0.36 to 0.62; Pseudomonadota: SMD − 0.26 to −0.68), confirming the robustness of these findings. Unlike the other two studies included in these analyses, this study exclusively enrolled patients with cryptogenic ischemic stroke. Given that oral microbiota profiles may differ across stroke subtypes, this inconsistency in inclusion criteria likely constitutes a key source of heterogeneity in the corresponding analyses.

### 3.7 Bias in publication

The funnel plot (Supplementary Figure 3) showed no significant evidence of publication bias.

### 3.8 Certainty of evidence

The GRADE assessment results show that, with the exception of Fusobacteriota, for which the certainty of evidence was very low (⊕○○○), the certainty of evidence for the remaining seven outcome measures was low (⊕ ⊕ ○○). Please refer to Supplementary Table 3 for the specific assessment results.

## 4 Discussion

To our knowledge, this meta-analysis represents the first systematic evaluation of *α*-diversity, *β*-diversity and taxonomic differences at the phylum and genus levels in the oral microbiota of stroke patients. The results showed increasing trends in the observed species and Shannon indices in the stroke group compared with healthy controls, while no significant differences were observed in the Chao1 and Simpson indices. However, these findings were not robust in sensitivity analyses. Furthermore, eight out of nine studies reported significant deviations in β-diversity among stroke patients, suggesting a marked alteration in the overall microbial community structure. Regarding microbial composition, meta-analysis showed that stroke patients had higher abundance of *Bacteroidota* (SMD = 0.36, 95% CI: 0.18–0.54, 3 studies; I^2^ = 44%). Descriptive synthesis suggested higher abundances of *Firmicutes, Spirochaetes,* and several genera (*Lactobacillus, Leptotrichia, Prevotella, and Fusobacterium*) in stroke patients, although these findings were not quantitatively validated and should be considered exploratory. In summary, the oral microbiome of stroke patients exhibits a disease-associated structural dysbiosis, rather than a simple increase in microbial abundance.

Oral dysbiosis may contribute to the pathophysiology of stroke through various mechanisms. Regarding direct pathways, opportunistic pathogens in the oral cavity and their toxic by-products can enter the bloodstream via damaged gingival epithelium, thereby promoting the formation of atherosclerotic plaques (Kiramira et al., 2024; Zhong et al., 2024). Oral bacterial DNA has been detected in cerebral thrombi, suggesting that these bacteria may be directly involved in thrombus formation (Gayo et al., 2025). Indirectly, oral dysbiosis may induce a state of chronic periodontitis, releasing inflammatory mediators into the systemic circulation and exacerbating traditional stroke risk factors such as hypertension and dyslipidaemia (Tran et al., 2026; Lima et al., 2026). Regarding the ‘oral-gut-brain axis’ concept, oral pathogens swallowed may influence gut microbiota and immune cell function, thereby intensifying neuroinflammatory responses following a stroke (Tran et al., 2026). Animal studies have further revealed that the salivary microbiota associated with periodontitis may exacerbate post-stroke neuroinflammatory responses by increasing the number of IL-17A-producing immune cells in the gut and promoting their migration to the brain (Chen et al., 2022). The aforementioned mechanisms provide a theoretical basis for further understanding the relationship between the oral microbiota and stroke; however, their specific value in stroke prevention and treatment requires further research and validation.

In this study, oral *α*-diversity in stroke patients showed an upward trend in certain indices (Observed species and Shannon index), consistent with several previous studies (Yao et al., 2025; Sun et al., 2023; Zhao et al., 2025; Wang et al., 2022; Yang, 2022). However, sensitivity analyses indicated that this result was not robust; upon exclusion of a single study, the effect size converged to zero and lost statistical significance. Therefore, the conclusion regarding elevated oral *α*-diversity in stroke patients should be regarded as an exploratory finding and interpreted with caution. Furthermore, no significant differences were observed in either the Chao1 or Simpson indices, and some previous studies also failed to detect statistical differences in α-diversity between stroke patients and healthy controls (Zheng et al., 2026; He et al., 2024; Chang, 2024; Huang, 2024; Manzoor et al., 2025; Manzoor et al., 2024), These inconsistent findings further suggest that the observed trend toward increased α-diversity should be interpreted with caution. This inconsistency may stem from differences between studies in terms of sample size, demographic characteristics, stroke subtypes, disease severity and the type of oral samples. With regard to *β*-diversity, the vast majority of studies included in this review reported significant differences between stroke patients and healthy controls; this finding is highly consistent with the results of several original studies (Yao et al., 2025; Sun et al., 2023; Zhao et al., 2025; Zheng et al., 2026; He et al., 2024; Huang, 2024; Yang, 2022; Manzoor et al., 2025), further supporting the view that the oral microbiome undergoes structural remodeling in stroke patients.

The *Firmicutes* and *Bacteroidetes* phyla are the dominant bacterial phyla in the oral cavity and gut, and are closely associated with a variety of diseases (Adnan et al., 2017; Grigor'eva, 2020; Ling et al., 2015). Based on a meta-analysis of three studies showed that the abundance of the *Bacteroidetes* phylum in the oral microbiota of stroke patients was higher than that in healthy controls (SMD = 0.36, 95% CI: 0.18–0.54, I^2^ = 44%), which is consistent with the findings of previous studies (Zhao et al., 2025; Wang et al., 2022; Chang, 2024; Huang, 2024). Furthermore, the *Firmicutes* phylum showed a trend toward higher abundance according to a descriptive meta-analysis (four out of six studies reported increased abundance); however, as this result has not been validated by a quantitative meta-analysis, it should be regarded as an exploratory finding. It is worth noting that most studies have reported a reduced abundance of the *Bacteroidetes* phylum in the gut microbiota of stroke patients (Hou et al., 2025; Bao et al., 2024; Tan et al., 2021), which contrasts with the findings in the oral cavity in this study. This discrepancy may reflect differences in the microenvironments of the oral cavity and the gut (such as oxygen partial pressure, pH and nutrient sources), leading to different response patterns in the microbiota of these two sites to the same disease state. Although the composition of the oral and gut microbiota is site-specific, one study included in this review detected genera such as *Streptococcus* and *Neisseria* in both the oral cavity and gut of stroke patients (Yao et al., 2025), suggesting a degree of overlap between the microbiota of these two sites. However, there is currently a lack of direct evidence regarding the specific direction of the association between oral and gut microbiota in stroke patients and the potential migration pathways; this requires verification in future through longitudinal cohort studies or strain tracing analyses.

Stroke patients often have comorbidities such as hypertension, heart disease or mental disorders, and commonly used medications—such as diuretics, antihypertensives, antidepressants and antipsychotics—often have anticholinergic effects, which can lead to reduced salivary secretion (Sasegbon and Hamdy, 2017; Malallah et al., 2018). Furthermore, stroke can exacerbate reduced salivary secretion by impairing the neural innervation of the salivary glands and oral sensory function (Maciejczyk et al., 2020; Krausch-Hofmann et al., 2019). Reduced salivary secretion lowers the partial pressure of oxygen in the oral cavity, creating favorable conditions for the proliferation of anaerobic bacteria. This review found that the abundance of anaerobic bacteria, such as *Spirochaetes*, in the oral cavity of stroke patients showed an increasing trend based on descriptive synthesis, which is consistent with the aforementioned microenvironmental changes. However, meta-analysis of *Fusobacteriota* showed no statistically significant difference between the two groups (3 studies; SMD = −0.10, 95% CI: −0.46 to 0.27, I^2^ = 65%). Of note, although descriptive synthesis suggested increased trends for both *Fusobacteriota* (phylum) and *Fusobacterium* (genus) in stroke patients, this was not confirmed by meta-analysis, indicating a discrepancy between descriptive synthesis and pooled estimates. This finding should be interpreted with caution and warrants further validation in larger studies. Nevertheless, given the well-established role of anaerobic bacteria as key pathogenic agents of periodontitis (Minty et al., 2019), and the recognition of periodontitis as an independent risk factor for stroke (Meng and Chen, 2025), the potential clinical relevance of anaerobic bacteria in stroke patients cannot be entirely excluded and merits further investigation.

This review found that, in a pooled analysis, the abundance of the *Proteobacteria* phylum in the oral cavity of stroke patients showed a downward trend (3 studies, SMD = −0.48, 95% CI: −1.02 to 0.06, I^2^ = 83%); however, the difference was not statistically significant and there was high heterogeneity, so this result should be interpreted with caution. A descriptive synthesis also supports this finding (3 out of 3 studies reported a reduction in abundance). Members of the *Proteobacteria* phylum are predominantly aerobic or facultative anaerobic bacteria, and are highly sensitive to reduced oxygen partial pressure. Stroke patients often present with periodontitis, and deepening of periodontal pockets can reduce local oxygen partial pressure (Loesche et al., 1983); this may be one of the reasons for the reduced abundance of the *Proteobacteria* phylum. However, previous Mendelian randomization studies have suggested that increased abundance of *Proteobacteria* in the blood or gut is associated with an elevated risk of large-artery atherosclerotic stroke (Li et al., 2021). The discrepancy in the direction of *Proteobacteria* changes between the oral cavity and other body sites may reflect site-specific microenvironmental differences (such as reduced oxygen partial pressure in the oral cavity), rather than systemic alterations in microbial abundance. Since the included studies did not simultaneously collect samples from multiple sites, this hypothesis could not be directly tested in the present study.

## 5 Limitations

Despite these intriguing findings, our study has several limitations. Firstly, as the vast majority of studies originated in China, it is questionable whether the results can be generalized to other populations. Secondly, all included studies were assessed using the NOS scale and were judged to be at low risk of bias (NOS score ≥7), indicating a high overall quality of the included studies, which enhances the reliability of the present study’s findings. Sensitivity analyses showed that, following the sequential exclusion of individual studies, the direction of most pooled effects did not change significantly; however, some results were sensitive to specific studies, suggesting that these findings should be interpreted with caution. Statistically significant heterogeneity was observed among the included studies, with potential sources including differences in dietary patterns, geographical context, disease inclusion criteria (e.g., treatment regimens, drug dosages, disease duration) and factors related to oral samples (e.g., collection site, sample type, time of collection). Due to the limited number of included studies, subgroup analyses could not be performed, which may introduce methodological bias. The high level of heterogeneity suggests that the precision of the pooled effect size is limited; therefore, the conclusions of this study should be regarded as exploratory findings rather than definitive conclusions. Nevertheless, we applied a random-effects model to estimate the effect size in order to reduce the impact of heterogeneity on the results. Thirdly, it should be noted that the use of various nucleic acid extraction methods and gene sequencing technologies (see Table 1) may lead to bias in the results. For example, compared with analyzing the V4 region alone, differences in *α*-diversity between groups may be more pronounced when analyzing the V3–V4 region. However, due to the limited number of relevant studies (two for the V4 region and three for the V3–V4 region), we were unable to conduct further subgroup analyses. Fourthly, in several studies, we manually extracted the required data from histograms, which may introduce another source of bias. However, this extraction process was thoroughly discussed and agreed upon by two reviewers. As this method was applied consistently throughout the study, the direction of statistical significance in between-group comparisons is unlikely to be substantially affected.

## 6 Conclusion

In summary, our findings reveal characteristic alterations in the oral microbiota of stroke patients (increased *α*-diversity in certain indices, significant deviation in *β*-diversity, and enrichment of *Bacteroidetes*). However, the observed increase in α-diversity should be interpreted with caution, as it was not robust in sensitivity analyses. The enrichment of *Bacteroidetes* was based on only three studies and should be considered exploratory. Findings for *Firmicutes, Spirochaetes*, and genera-level taxa were based on descriptive synthesis and have not been quantitatively validated, and thus should also be regarded as exploratory. These findings provide preliminary insights into post-stroke oral dysbiosis and offer a theoretical basis for developing targeted oral care strategies, but require validation in future large-scale prospective studies.

## Data Availability

The original contributions presented in the study are included in the article/Supplementary material, further inquiries can be directed to the corresponding author.
