# Correction: Diversity and taxonomic differences in the oral microbiota of stroke patients: a systematic review and meta-analysis

**DOI:** 10.3389/fmicb.2026.1941491

**Published:** 2026-07-31

**Authors:** Yinlian Chen, Yunxue Tian, Yingju Jin, Xueqin Wu, Xiaomei Li, Wei Du, Wuanqin Li, Juan Li

**Affiliations:** 1 School of Nursing, Zunyi Medical University, Zunyi, China; 2 School of Nursing, Guizhou Medical University, Guiyang, China; 3 School of Nursing, Guizhou University of Traditional Chinese Medicine, Guiyang, China; 4 Department of Nursing, Guizhou Provincial People's Hospital, Guiyang, China

**Keywords:** stroke, oral microbiota, dysbiosis, systematic review, meta-analysis

The affiliation “School of Nursing, Guizhou University of Traditional Chinese Medicine, Guiyang, China” was omitted from the published article. This affiliation has been added for authors Yingju Jin, Xueqin Wu, Xiaomei Li, Wei Du and Wuanqin Li as affiliation 3. The corrected author and affiliation list is as follows:

Yinlian Chen^1†^, Yunxue Tian^2†^, Yingju Jin^3†^, Xueqin Wu^3^, Xiaomei Li^3^, Wei Du^3^, Wuanqin Li^3^ and Juan Li^4^^*^

^1^School of Nursing, Zunyi Medical University, Zunyi, China

^2^School of Nursing, Guizhou Medical University, Guiyang, China

^3^School of Nursing, Guizhou University of Traditional Chinese Medicine, Guiyang, China

^4^Department of Nursing, Guizhou Provincial People's Hospital, Guiyang, China

The original version of this article has been updated.

