# Supplementary material for: Diversity and taxonomic differences in the oral microbiota of stroke patients: a systematic review and meta-analysis
Source: Front Microbiol. 2026 Jul 15;17:1874193. doi: 10.3389/fmicb.2026.1874193 (PMC13416613; doi:10.3389/fmicb.2026.1874193)
Supplement: Supplementary file 1 [file Supplementary_file_1.docx]

***Supplementary Material***

**Oral microbiota in patients with stroke: a systematic review and meta-analysis**

Yinlian Chen^1^, Yunxue Tian^2^, Yingju Jin^3^, Xueqin Wu ^3^, Xiaomei Li ^3^, Wei Du ^3^, Wuanqin Li ^3^，Juan Li ^4*^

*** Correspondence:**

**Juan Li E-mail:694807055@qq.com**

**Supplementary table 1. Search strategies of Wanfang.**

| **Number** | **Search Strategies** |
| --- | --- |
| #1 | **主题:(卒中 or 急性卒中 or 脑卒中 or 脑中风 or 脑血管意外 or CVA or CVAs or 脑血管中风 or 中风；急性 or 急性脑血管意外 or 急性脑卒中 or 中风 or 缺血性脑卒中 or 出血性脑卒中)** |
| #2 | **主题:(口腔微生物组 or 口腔微生物群 or 口腔菌群 or 口腔微生物 or 口腔微生态 or 牙菌斑微生物 or 唾液菌群)** |
| #3 | **#1 AND #2** |

**Supplementary table 2. Search strategies of PubMed databases.**

| **Number** | **Search Strategies** |
| --- | --- |
| #1 | **(((((((((((((((((((((((((((((stroke) OR (strokes)) OR (Cerebrovascular Accident)) OR (Cerebrovascular Accidents)) OR (CVA (Cerebrovascular Accident))) OR (CVAs (Cerebrovascular Accident))) OR (Cerebrovascular Apoplexy)) OR (Apoplexy, Cerebrovascular)) OR (Vascular Accident, Brain)) OR (Brain Vascular Accident)) OR (Brain Vascular Accidents)) OR (Vascular Accidents, Brain)) OR (Cerebrovascular Stroke)) OR (Cerebrovascular Strokes)) OR (Stroke, Cerebrovascular)) OR (Strokes, Cerebrovascular)) OR (Apoplexy)) OR (Cerebral Stroke)) OR (Cerebral Strokes)) OR (Stroke, Cerebral)) OR (Strokes, Cerebral)) OR (Stroke, Acute)) OR (Acute Stroke)) OR (Acute Strokes)) OR (Strokes, Acute)) OR (Cerebrovascular Accident, Acute)) OR (Acute Cerebrovascular Accident)) OR (Acute Cerebrovascular Accidents)) OR (Cerebrovascular Accidents, Acute)) OR (("Stroke"[Mesh]))** |
| #2 | **(((((((((((((((((Microbiota) OR (Microbiotas)) OR (Microbial Community)) OR (Community, Microbial)) OR (Microbial Communities)) OR (Microbial Community Composition)) OR (Community Composition, Microbial)) OR (Composition, Microbial Community)) OR (Microbial Community Compositions)) OR (Microbiome)) OR (Microbiomes)) OR (Human Microbiome)) OR (Human Microbiomes)) OR (Microbiome, Human)) OR (Microbial Community Structure)) OR (Community Structure, Microbial)) OR (Microbial Community Structures)) OR (Oral Microbiota)** |
| #3 | **((((((((((((Oral) OR (Saliva)) OR (Salivas)) OR (Mouth)) OR (Cavitas Oris)) OR (Oral Cavity)) OR (Cavity, Oral)) OR (Vestibule of the Mouth)) OR (Vestibule Oris)) OR (Oral Cavity Proper)) OR (Cavitas oris propria)) OR (Mouth Cavity Proper)) OR (Oropharynx)** |
| #4 | **#1 AND #2 AND #3** |

**Figure 1**

A
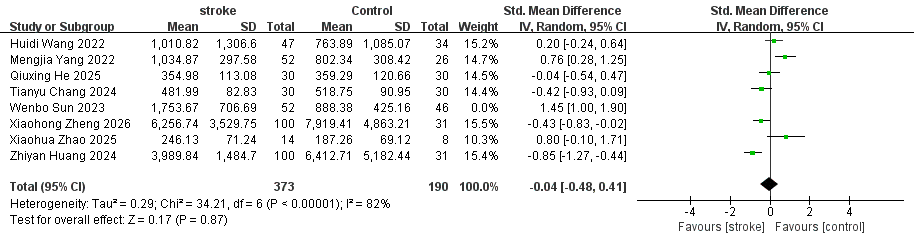


B
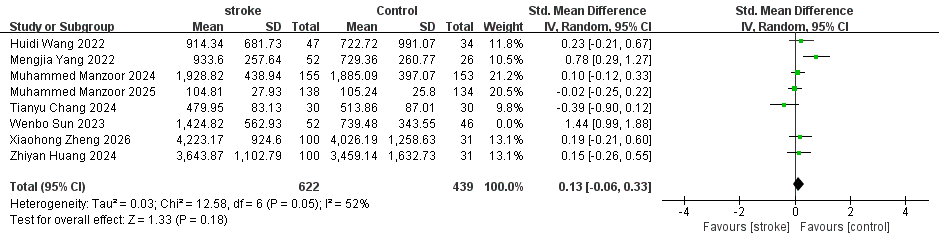


C
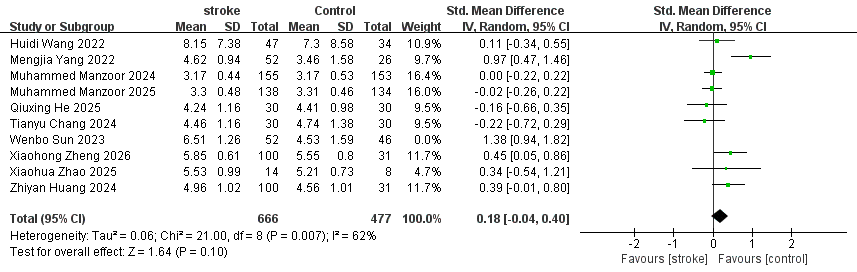


**Supplementary Figure 1.** Forest plot of sensitivity analysis for α diversity

Forest plot of sensitivity analysis for Chao1**(A)**.

Forest plot of sensitivity analysis for Observed species**(B)**.

Forest plot of sensitivity analysis for Shannon**(C)**.

**Figure 2**

A
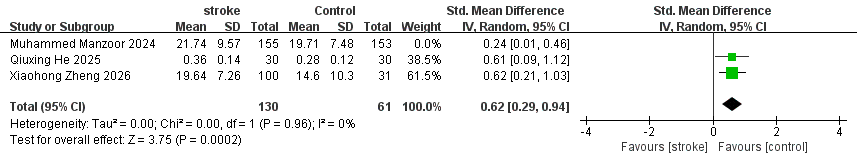


B
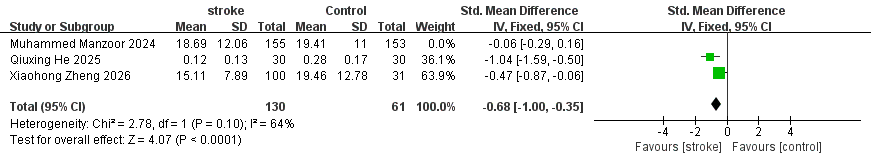


**Supplementary Figure 2.** Forest plot of sensitivity analysis for the phyla Bacteroidota and Pseudomonadota

Forest plot of sensitivity analysis for the phyla Bacteroidota **(A).**

Forest plot of sensitivity analysis for the phyla Pseudomonadota **(B).**

**Figure 3**

| A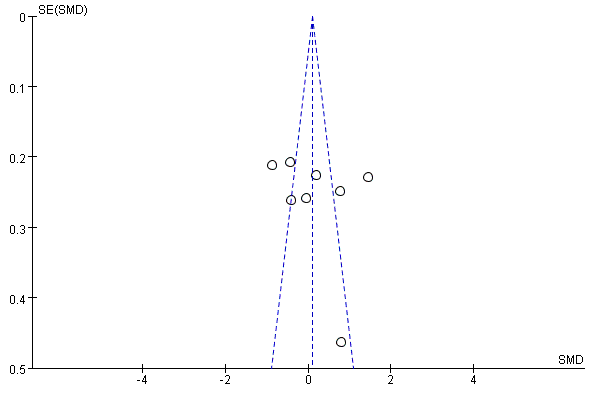 | B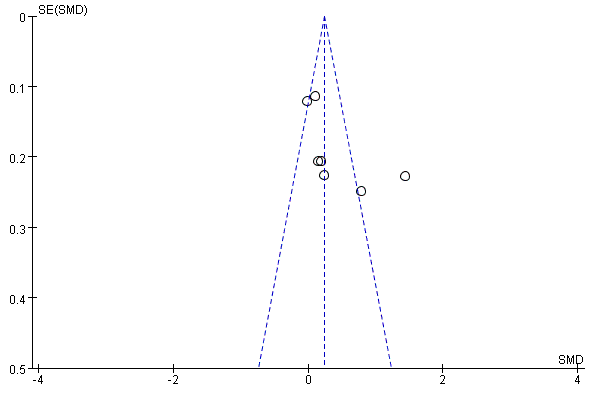 |
| --- | --- |
| C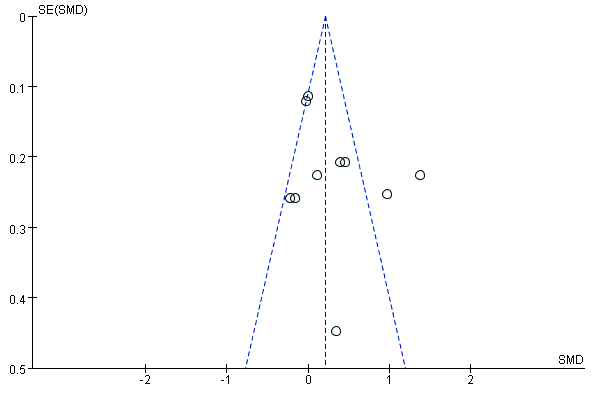 | D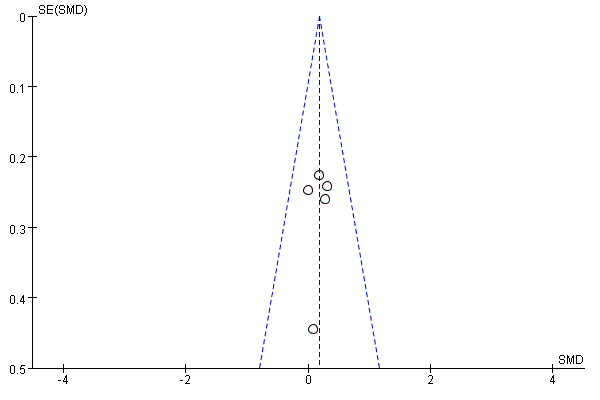 |
| E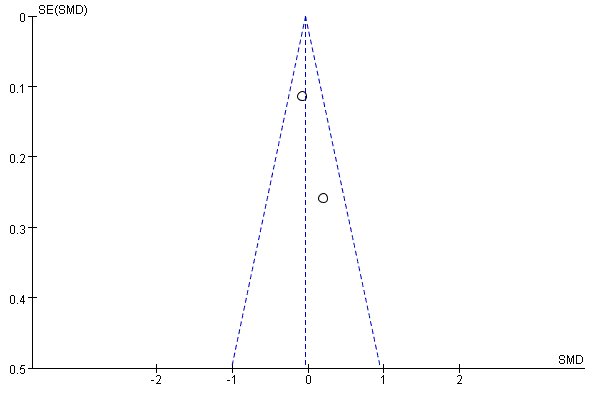 | F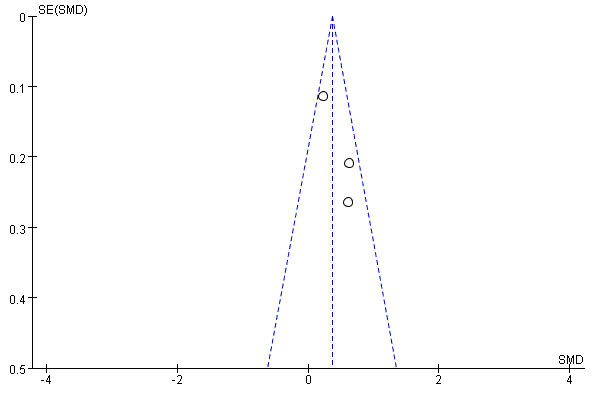 |
| G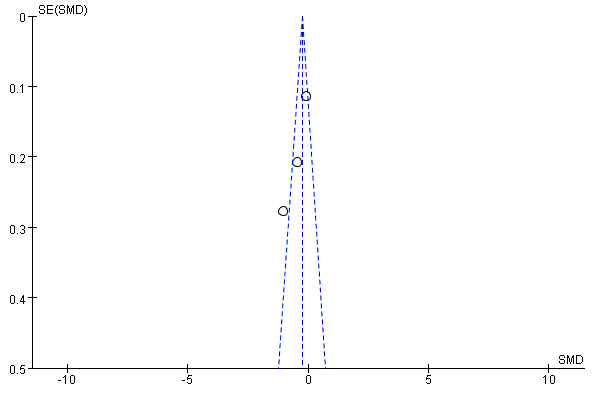 | H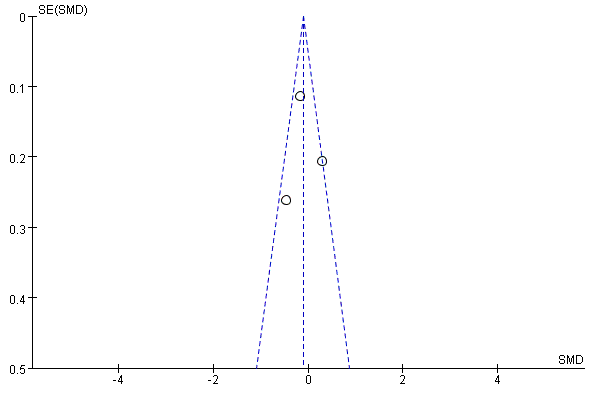 |

**Supplementary Figure 3.** Funnel plot for assessment of publication bias

Funnel plot for assessment of publication bias for Chao 1 **(A).**

Funnel plot for assessment of publication bias for Observed species **(B).**

Funnel plot for assessment of publication bias for Shannon **(C).**

Funnel plot for assessment of publication bias for Simpson **(D).**

Funnel plot for assessment of publication bias for Actinomycetota **(E).**

Funnel plot for assessment of publication bias for Bacteroidota **(F).**

Funnel plot for assessment of publication bias for Pseudomonadota **(G).**

Funnel plot for assessment of publication bias for Fusobacteriota **(H).**

**Table 3. GRADE certainty of evidence rating for differences in oral microbiota between stroke patients and healthy controls**

| Outcome | Studies (n) | Research design | Bias risk | Inconsistency | Indirectness | Imprecision | Number of patients (cases)  S C | | Differences between groups [MD（95%CI)] | Effect (SMD,95%CI) | Importance |
| --- | --- | --- | --- | --- | --- | --- | --- | --- | --- | --- | --- |
| Chao 1 | 8 | Case-control | Not serious | Extremely serious① | Not  serious | Serious② | 425 | 236 | 0.17[-0.40,0.73] | ⊕⊕○○ Low | Important |
| Observed species | 8 | Case-control | Not serious | Serious③ | Not  serious | Not serious | 644 | 455 | 0.39[0.05, 0.72] | ⊕⊕○○ Low | Important |
| Simpson | 5 | Case-control | Not serious | Not serious | Not  serious | Not serious | 179 | 128 | 0.18[-0.05,0.41] | ⊕⊕○○ Low | Important |
| Shannon | 10 | Case-control | Not serious | Serious④ | Not  serious | Not serious | 718 | 523 | \| 0.31[0.02,0.61] \| \| --- \| | ⊕⊕○○ Low | Important |
| Actinomycetota | 2 | Case-control | Not serious | Not serious | Not  serious | Not serious | 185 | 183 | -0.04[-0.24,0.17] | ⊕⊕○○ Low | Important |
| Bacteroidota | 3 | Case-control | Not serious | Not serious | Not  serious | Not serious | 285 | 214 | 0.43[0.14, 0.71] | ⊕⊕○○ Low | Important |
| Pseudomonadota | 3 | Case-control | Not serious | Serious⑤ | Not  serious | Not serious | 285 | 214 | -0.26[-0.44,-0.07] | ⊕⊕○○ Low | Important |
| Fusobacteriota | 3 | Case-control | Not serious | Serious⑥ | Not  serious | Serious⑦ | 285 | 214 | -0.10[-0.46,0.27] | ⊕○○○ Very low | Important |

Note: ① I²=91%; ② 95%CI crossed the null with wide width; ③ I²=84%; ④ I²=82%; ⑤ I²=83%; ⑥ I²=65%; ⑦ 95%CI crossed the null with wide width. The starting certainty level was low (observational studies). The final certainty was determined after downgrading assessments according to the GRADE framework.
